# From Basic Radiobiology to Translational Radiotherapy

**DOI:** 10.3390/ijms232415902

**Published:** 2022-12-14

**Authors:** Piyawan Chailapakul, Takamitsu A. Kato

**Affiliations:** Department of Environmental and Radiological Health Sciences, Colorado State University, Fort Collins, CO 80523, USA

The Special Issue, entitled “From basic radiobiology to translational radiotherapy”, highlights recent advances in basic radiobiology and the potential to improve radiotherapy in translational research. A major goal of radiotherapy is controlling tumor volume with minimal effects on surrounding normal tissues. To achieve this, basic radiation responses in normal cells and cancer cells must be well-understood. Although radiation can kill tumors with a high enough dosage, we need to spare normal tissue. General sensitization and protection are important findings in current scientific research; however, in clinical research, normal tissue specific protection and the specific sensitization of tumor cells are important. Nine papers are included in this Special Issue. This editorial briefly introduces these papers, the challenges that they address in radiotherapy, and strategies to manage these challenges (Table 1). Four research papers focus on enhancing the radiosensitivity of cancer cells. One research paper investigated proteomic profiles to prevent radiation-induced apoptosis. Four review papers summarized recent advances in radioresistant tumors, novel targets for brain radiotherapy, and high LET radiation therapy.

In cell molecular biology, intrinsic cellular radiation responses and sensitivity are significantly associated with DNA damage responses (DDRs), including DNA repair and cell cycle checkpoint activation [1]. The alteration of these DDRs leads to a hypersensitivity to radiation. Additionally, traditional radiation is less effective in hypoxic cells [2,3]. Even for an in vitro cell culture experiment, we do not need to consider hypoxic effects since tumors, especially radioresistant tumors, contain a hypoxic fraction, and this is a major problem for radiotherapy. Highly apoptotic tumors are often radiosensitive but radiation-induced apoptosis causes the major problem of normal tissue injury after radiotherapy. Cell-to-cell communication is an important biological response in vitro and in vivo. Pazzaglia et al. showed radiation-induced plasma miRNA and proteomic prolife changes in mice brains [4]. Whole-body irradiation-induced exosomal miRNA in plasma injection prevented radiation-induced apoptosis in mice. Their findings show that irradiated plasma reduces radiation injury and may contribute to the therapeutic application of exosome-based radiation injury treatment in the central nervous system (CNS). Exosomes can be mediators of intercellular communication and affect radiation responses in the tissue microenvironment. One of the most interesting lines of research is to inRvestigate the potential radiation injury protection from probiotics used for intestinal complications. Zeng et al. show that gut microorganisms affect radiation-induced injury response in the intestine [5]. The mice had gut microbiotas modulated by antibiotic cocktail and fecal microbiota transplantation; then, radiation-induced intestinal injury after total abdominal irradiation was observed, and these treatments can affect the social hierarchy. They suggested *L. murinus* and *A. muciniphila* might be potential probiotics against radiation toxicity. 

Radiosensitization is a strategy to increase cellular or tumor radiosensitivities. It can be achieved by halogenated pyrimidines, DNA repair inhibitors, and hypoxic sensitizers [6]. Radioresistance is major problem for the efficacy of radiotherapy. Radioresistance is caused by molecular changes at the cellular level and tumor microenvironmental situations so it affects to response to radiation. In each case, specific targeting strategies can overcome radioresistant tumors. In their review, Busato et al. summarized the biological factors that affect radioresistance [7]. Radioresistant cancer cells might have abnormal molecular features in terms of genetics and epigenetics. Additionally, the tumor microenvironment is modified by the presence of malignancies, stromal cells, cancer stem cells, tumor stem cells, tumor metabolism, and oxygen concentration. To increase the therapeutic efficacy of radioresistant tumors, many radiosensitizers were developed to make cancer cells more sensitive to radiation therapy, e.g., hypoxic cell radiosensitizers, radiosensitizing chemotherapy agents, nanoparticles, and immunomodulators. Furthermore, high-linear-energy-transfer radiotherapy, stereotactic body radiation therapy, and FLASH radiotherapy are used to kill some of these radioresistant agents. Since a critical target of ionizing radiation is DNA, the damage response is important in radiosensitivity. Typically, radiosensitive mutants are isolated, genes are cloned, and their functions are investigated using molecular biology techniques [8]. Recent advances in molecular techniques allow genome-wide screening technologies to be developed to explore genes that impact individual radiosensitivity. Tamaddondoust et al. summarized the most recent studies that have used a whole-genome analysis by DNA microarray analysis, RNA sequencing, siRNA library and CRISPR-Cas9, identifying genes that impact radiosensitivity [9]. Zhao et al. demonstrated a novel molecular target for breast cancer radioresistance [10]. Using gene expression profiles from breast cancer patients’ samples, they found that higher GDF15 expression is associated with poor prognosis and cellular radioresistance. Their study suggests that GDF15 may induce epithelial-mesenchymal transition (EMT) and tumor stemness. Since cancer stem cells can be radioresistant cells [11], the inhibition of GDF15 may reduce cancer stem cell population, improve local tumor control, and enhance the radiotherapeutic efficacy of radiotherapy. As an alternative approach to the treatment of pathological vasculature and stereotactic radiosurgery, vascular targeting with precision thrombosis is used for brain arteriovenous malformations. Currently, externalized phosphatidylserine and alpha-B-crystallin are used for radiation-induced surface markers for in vitro testing. Faqihi et al. demonstrated the vascular targeting agents (VTAs), which consist of an anti-PDCE2 antibody (E2 subunit pyruvate dehydrogenase: PDCE2) linked to a thrombin called “coaguligand” as an irradiated surface marker, and conducted selective drug delivery in a parallel-plate flow system [12]. Anti-PDCE2 antibody was conjugated with fluorophores and thrombin. Using this conjugated antibody, selective thrombosis occurred in the irradiated area due to accumulated thrombin. This precision thrombosis could be used for the advancement of radiation-guided drug delivery to animal models. 

We know several tumors are difficult to treat, such as malignant brain tumors, melanoma, pancreatic cancer, and lung cancers. Additionally, brain glioma is difficult to treat since it often metastasizes, making surgery challenging. Chemotherapy can be difficult due to the poor penetration of chemotherapeutic agents through the blood–brain barrier (BBB). Radiation is one of a modality to brain tumors, and gamma-knife or cyber-knife treatments can concentrate doses to tumors and spare normal brain tissue. However, the brain is an essential organ, and its radiation responses and normal tissue injury can severely affect patients’ quality of life (QoL). Microglia acts as a brain immune system in the irradiated brain and, removing damaged brain cells but also inducing inflammation. Persistent activation of microglia may contribute to radiation-induced brain injury, which is one of the most major problems for radiotherapy in the brain [13]. Liu et al. summarized molecular mechanisms and endpoints of radiation-induced microglia activation and brain injury [14]. They also summarized recent studies on the protective targets of brain injury. However, its mechanisms, aging, and the aspect of gender must be investigated in preclinical studies. Adjusting and managing abnormal neurons using balanced microglial phagocytosis might decrease side effects. Whole-brain radiotherapy is established for the treatment of patients with multiple brain metastases. However, the treatment of patients with high-grade glioma is challenging due to the radiation toxicity to normal brain tissue. Jaekel et al. investigated high-dose microbeam irradiation as a simultaneous integrated boost modality combined with whole-brain radiotherapy [15]. Microbeam radiotherapy is a novel therapeutic approach to utilizing high-dose-rate radiotherapy with a spatial dose fractionation in the micrometer range. No clear side effects were observed in irradiated mice. In vitro research shows that microbeam radiotherapy followed by whole-brain radiotherapy has stronger cell-killing effects than an irradiation scheme in reverse order. Since microbeam radiotherapy improves differential effects between normal cells and tumor cells, treatment doses can be increased, while the functioning of normal tissues is protected.

Non-small-cell lung cancer is a major cancer with a high incidence and high mortality ratio. High-grade tumors are often difficult to resect and treat with radiotherapy. Normal tissue damages are some adverse effects of radiotherapy, but hadron radiotherapy, including proton radiotherapy and carbon ion radiotherapy, can minimize normal tissue doses and reduce unwanted late effects due to a superior dose distribution within a patient’s body. Liang et al. summarized cellular responses of carbon ion irradiation and recent clinical data for NSCLC [16]. Carbon ion radiotherapy uses accelerated carbon ions. It has high LET radiation properties, higher relative biological effectiveness, and lower oxygen enhancement ratio resulting in better tumor controls. Additionally, CIRT can inhibit potential angiogenesis and metastasis because of its high tumor-killing ability. In addition, the synergistic effects of CIRT with the signaling pathway modification can overcome NSCLC cells and effectively function under hypoxic conditions. Currently, single fraction high-dose CIRT with a good outcome has been used, but it is needed to balance adverse reactions. However, some challenges are still present in terms of sample size, the application field and cost. Considering the poor prognosis of the recurrence of NSCLC tumors, treatment should be combined with other therapies. Radiation pneumonia and adverse reactions are necessary to consider this effect.

**Table 1:** Challenges of radiotherapy and how to solve actual treatment problems are discussed in this Special Issue.

Challenges of Radiotherapy	Actual Problems	Potential Strategies
Radioresistant tumors	Radioresistant hypoxia	Hypoxia cell sensitizer [7], High-LET radiation [16]
	Radioresistant cancer cells	Modulating DDR for radiosensitization [9]
	Cancer stem cells	Reducing stemness by modulating responsive genes [10]
	Combination with chemotherapeutic agents	Efficient drug delivery system [12]
Normal tissue damage	Intestine damage	Modulation of microbiota to reduce toxicity [5]
	Brain injury	Modulating exosomes [4], Modulation of activated microglia [14], Combination with high-dose microbeam [15]
